# Employment Status Among U.S. Military Veterans With Traumatic Brain Injury: Mediation Analyses and the Goal of Tertiary Prevention

**DOI:** 10.3389/fneur.2019.00190

**Published:** 2019-03-15

**Authors:** Laraine Winter, Helene Moriarty, Keith Robinson

**Affiliations:** ^1^Corporal Michael J. Crescenz VA Medical Center, Philadelphia, PA, United States; ^2^M. Louise Fitzpatrick College of Nursing, Villanova University, Villanova, PA, United States

**Keywords:** traumatic brain injury, military veterans, employment, depression, physical functioning, pain, tertiary care

## Abstract

For most individuals with traumatic brain injury (TBI), the ability to work is crucial to financial and psychological well-being. TBI produces a wide range of cognitive, physical, emotional, and interpersonal impairments that may undermine the ability to work. Employment is therefore a primary goal of TBI rehabilitation and has been the focus of extensive research. Although this literature has identified predictors of employment outcomes, few studies have examined the mechanisms that underlie these associations. Mediation analysis can identify these mechanisms, provide a more nuanced view of how predictors jointly affect rehabilitation outcomes, and identify predictors that, if treatable conditions, could be useful targets for tertiary prevention. Such efforts are aimed at reducing long-term impairments, disability, or suffering resulting from the injury. The study sample comprised 83 U.S. military veterans with TBI who had participated in a larger rehabilitation study and were interviewed in their homes. Bivariate tests revealed significant associations of employment with pain, cognitive functioning, self-rated health, depressive symptoms and physical functioning; the latter variable was operationalized in two ways—using the Patient Competency Rating Scale and the SF–36V physical functioning subscales. Because these physical functioning measures were highly intercorrelated (*r* = 0.69, *p* < 0.0001), separate regression models were conducted. In the hierarchical binary logistic regression models, predictors were entered in order of modifiability, with comorbidities (pain) entered in block 1, physical health/functioning sequelae in block 2, and depressive symptoms in block 3. In the regression using the SF-36V measure of physical functioning, pain's effect was mediated by the physical functioning/health predictors, with only physical functioning emerging as significant, but this effect was itself mediated by depressive symptoms. In the regression using the PCRS physical-function measure, only depressive symptoms emerged as a mediator of other effects. Findings underscore the central role of depression in the employment status of veterans with TBI, suggesting that negative effects of other problems/limitations could be mitigated by more effective treatment of depression. Thus, for many with chronic TBI who live with vocational limitations, outcomes may improve with lower depression. Findings argue for the wider use of mediation approaches in TBI research as a means of identifying targets for tertiary prevention of poor outcomes.

## Introduction

Traumatic brain injury (TBI) may produce a wide range of cognitive, emotional, interpersonal, and physical sequelae, (1–7) any of which may impair the ability to work. An important goal of rehabilitation for most individuals with TBI, work is crucial to financial and psychological well-being (8–10). The importance of employment is attested to by an extensive research literature documenting the impact of employment problems and identifying predictors of employment status, return to work (RTW), and employment stability (11–13).

Most studies of predictors of employment outcomes have used some form of multiple regression to identify those that contribute independently. Certain variables have emerged fairly consistently as predictors of employment: younger age at injury (14, 15), White race and non-Hispanic ethnicity (16–18), higher educational level (19), pre-injury unemployment (20), higher pre-injury occupational status (12, 14, 15); lower injury severity (15, 21, 22); higher Glasgow Coma Scale scores (19); higher cognitive function (12, 19, 23), everyday functioning (which may include physical, cognitive, emotional, interpersonal, or other dimensions) (11, 12, 24–27); and lower depression (7, 14, 28–38). Relatively neglected have been closer examinations of the interrelationships among these variables using mediation analyses. The use of mediation methods such as hierarchical multiple regression or structural equation modeling can provide a more nuanced view of how predictors jointly affect employment outcomes by identifying the mechanisms that underlie predictors' associations with employment outcomes (39). A mediator is an intervening variable that explains the reason for a relationship between an independent and dependent variable. It “carries the effect,” indicating that an independent variable leads to a change in the mediator variable, which in turn leads to a change in the dependent variable (40).

Findings from mediation analyses can have practical implications for clinical practice, especially when the mediators represent modifiable conditions. These implications relate especially to tertiary prevention of TBI outcomes. Whereas primary prevention concerns preventing injury or disease, and secondary prevention concerns detecting them in their earliest stages and intervening to slow or stop their progression, the aim of tertiary prevention is to reduce long-term impairments, disability, and suffering resulting from them (41). Tertiary prevention efforts strive to soften the impact of an ongoing illness or injury that has lasting effects. Focusing on strategies to manage long-term health problems, tertiary prevention commonly includes chronic disease self-management programs and vocational rehabilitation programs (42).

Tertiary prevention has received relatively little research attention in TBI rehabilitation, which has overwhelmingly focused on its early post-injury phases (43–45). Yet, TBI sequelae often persist for years, and TBI is increasingly viewed as a chronic illness (20, 46–53). Its sequelae may undermine quality of life, community integration, and cognitive, emotional, and vocational functioning. Interventions for the chronic stage, after symptoms have stabilized, have been relatively neglected, leaving many individuals to deal with chronic TBI symptoms on their own (54). Therefore, tertiary prevention should be an important focus for TBI.

A treatable condition that is identified as a mediator of other effects may be a promising target for intervention efforts, because even if the predictor whose effect is mediated does not itself improve, treating the mediating condition may improve long-term outcomes. For example, the finding that depressive symptoms mediate the effect of post-traumatic stress disorder (PTSD) on community reintegration (55) would suggest that, even when PTSD has remediated as much as possible, community reintegration could be further improved by treating the depression. This is a tertiary prevention strategy.

Mediation analyses may therefore help identify targets for tertiary prevention efforts. To be a target for tertiary prevention, a predictor would have to represent a treatable condition and be shown to mediate effects of predictors entered previously. Thus, if a predictor is found to attenuate the effect of a previously-significant variable on an outcome, that mediating predictor may serve as a worthwhile rehabilitation focus. An analysis to test mediation would therefore order the variables' entry by degree of modifiability. Background characteristics like TBI severity and time post injury, would be entered first. Comorbidities, which are also background characteristics and may or may not be modifiable, would be entered next. Prominent among these would be PTSD (56, 57) and pain (57), both prevalent in veteran populations and highly comorbid with each other and with TBI. Entered next would be TBI sequelae. These commonly include limitations in cognitive, physical, emotional, and interpersonal functioning; and symptoms such as tinnitus and photosensitivity; and psychiatric disorders. Because psychiatric disorders are likely to be distinct from physical sequelae, these would be entered in a separate step.

Among psychiatric sequelae of TBI, depression is the most common and often has serious consequences for persons with TBI (35). It can interfere with motivation, diminish the ability to think or concentrate, produce feelings of pessimism and futility, and cause fatigue, loss of energy, and other somatic symptoms (53)—symptoms that can impair the ability to work. Depression has also been shown to mediate effects of other predictors on important outcomes in TBI, as mentioned earlier, mediating effects of both physical functioning and PTSD on community reintegration (CR) (defined as the extent to which the individual participates in activities with family, friends, and community) (58). Depression also mediates effects of insomnia on suicide risk among military veterans with or without TBI (59). In other (non-TBI) clinical populations, depression has been shown to mediate effects of severity, pain, or other predictors on rehabilitation outcomes such as functioning, quality of life, and community integration (60–63). Yet, depression is highly treatable (64). Among TBI patients, the most common depression treatments have been medication, especially the selective serotonin reuptake inhibitors (65–67), and cognitive behavioral therapy (CBT) (68–72). These considerations would argue for an analytic strategy of entering the depression variable separately from physical health and functioning in mediation models.

The present study examined predictors of employment status in a group of U.S. military veterans with TBI. These were outpatients at a U.S. Veteran Affairs medical rehabilitation clinic. Military TBI is known to differ from TBI in civilian populations in several respects. Multiple TBIs, high-energy explosives, and blast injuries are more common in military TBI (73). In addition, combat injuries are less likely to be diagnosed promptly and more likely to rely on self-reports (74). These features complicate efforts to characterize the severity of individual injuries, as might be done routinely after a civilian TBI event. Emotional distress tends to be greater in military TBI and comorbid mental health symptoms and conditions more prevalent. High rates of comorbidity among TBI, PTSD, and depression and their overlapping signs and symptoms complicate attribution to TBI or PTSD (75). Service members returning from military deployment may have more difficulty with community reintegration (56, 76).

In addition, within military TBI, a further distinction can be drawn between combat- and noncombat-related TBIs. In non-combat TBIs, mechanisms of injury such as vehicular crashes and falls may be similar to those in a civilian population (although crashes and falls also occur during combat). But most study participants had sustained multiple TBIs, some during combat (including many blast injuries) and others in non-combat situations. These consideration complicate efforts to classify TBI mechanisms.

Veterans in the present study had predominantly mild TBI (mTBI) with persistent post-concussive symptoms or mild-moderate TBI, and in this respect the study contrasts with most previous studies, which have tended to study more severe TBI. Yet mTBI accounts for more than half of cases worldwide (77), and mild to moderate TBI constitutes 82.7 percent of cases (78). Within the U.S., mTBI represents 80–90 percent (79). MTBI causes long-term mental and physical health consequences in a sizable minority of patients (80–83). A 2012 VA systematic review on complications of mTBI in veterans and military personnel estimated that 10–20 percent experience ongoing post-concussive symptoms (84).

The present study investigated potential mediation of predictors' effects on employment status. Hierarchical binary logistic multiple regression was used, with the order of entry determined by modifiability from least to most. The purpose of testing for mediation was to identify treatable conditions as potential targets for rehabilitation, especially for tertiary prevention.

The study was innovative in several respects. It used a mediation approach to seek targets for rehabilitation efforts toward tertiary prevention goals. It also utilized a less severely injured sample compared to most studies of employment outcomes and a military veteran population, who are also understudied in the area of the employment outcomes.

## Methods

### Design

This was a secondary analysis of data collected in a randomized controlled trial that evaluated the efficacy of an in-home intervention (the Veterans In-home Program) for U.S. military veterans with TBI and their family members (55, 85). Data reported in the present study were collected during the baseline interview with veterans, prior to randomization.

### Sample

Study participants were 83 veterans with TBI recruited from the Corporal Michael J. Crescenz Veterans Affairs (VA) Medical Center outpatient Rehabilitation Medicine Service. Since 2017, the VA has screened veterans of the Afghan and Iraq wars for TBI, evaluated those screening positive, and referred them to the Rehabilitation Medical Service. In addition, veterans from earlier war cohorts may be referred to this service by primary care, psychiatry, and other specialty providers. All study participants had recently received a TBI evaluation and TBI diagnosis through this service and were reporting current TBI-related symptoms (All had been screened for PTSD as well.). TBI etiology could be combat (e.g., blast exposure) or non-combat events such as vehicular crashes, falls, or equipment accidents, as explained above. Study participants were recruited using a letter of invitation mailed to eligible veterans, followed by a phone call that further described the study and confirmed the veteran's study eligibility, and determined his/her willingness to participate. Inclusion criteria included residence in the Philadelphia, Pennsylvania metropolitan region, diagnosis of TBI at the Polytrauma Program, post-deployment from the Vietnam War era to the present, ability to speak English, meeting VA Polytrauma Systems of Care criteria for TBI (86), and having a family member or partner living with him/her or living within close proximity and willing to participate in the study.

### Measures

#### Sociodemographic Characteristics

The interview provided information on veterans' age, race, sex, Hispanic ethnicity, religious affiliation, financial difficulty (87), years of education, marital status, number of years married, number of children, and employment status. Because only five veterans were found to be employed part-time, employment status was defined as employed part- or full-time vs. not employed. No one had voluntarily retired.

#### Military and Injury-Related Characteristics

Electronic medical records from the VA Computerized Patient Record System (CPRS) provided background information augmenting interview data. This information encompassed the veteran's war cohort, number of years since most recent TBI (time post injury), source of injury, number of TBIs, and comorbidities (posttraumatic stress disorder [PTSD], pain, tinnitus, and photosensitivity). PTSD was defined as the presence or absence of a PTSD diagnosis documented as active in CPRS during the study period. All U.S. veterans receiving services at the medical rehabilitation clinic are screened for PTSD.

TBI severity was determined using the VA/DOD Clinical Guidelines for Management of Concussion/Mild Traumatic Brain Injury (mTBI) (86). A physician with rehabilitation medicine expertise (K.R.) reviewed the data in the electronic records to determine TBI severity. The diagnosis of TBI in these military personnel was based on several factors, including exposure to one of several events that could induce cerebral damage, the persistence of clinical symptoms and signs indicating that a brain injury may have occurred, and findings on brain imaging, either computerized tomography or brain MRI. As expected in TBIs classified in the mild and mild-to-moderate ranges of severity, brain imaging often can be reported as having no structural damage. But this does not preclude the diagnosis of TBI. For analytic purposes, severity was dichotomized into mild vs. moderate to severe.

#### Health and Functioning: Short Form Health Survey-36 Veteran Version (SF-36V)

The SF-36 is widely used for monitoring and assessing care outcomes in adult patients (88). It has been modified for use in VA ambulatory care patient populations (the SF-36V) (89, 90) and has demonstrated strong reliability and validity. The SF-36V consists of eight physical and mental health concepts. The present study utilized only the physical health/functioning domains: physical functioning limitations (e.g., limitations in lifting groceries), pain intensity, extent of pain's interference with everyday functioning, and self-rated health.

#### Pain

Pain is measured in terms of intensity and extent of interference with normal work. The intensity question uses a 6-point scale, from 0 (none) to 5 (severe), and the interference question uses a 5-point scale, from 0 (not at all) to 4 (extremely). Therefore, raw scores were converted to *z*-scores. Because the two pain items were highly correlated (*r* = 0.76, *p* < 0.0001), a mean score was computed and used as a pain index (Cronbach's alpha = 0.86). Higher scores indicate worse pain.

#### Physical Functioning

The physical functioning subscale assessed extent of limitations in 10 activities, each item followed by a 3-point response format from 0 (not at all limited) to 2 (limited a lot). Thus, higher scores indicate worse functioning. Cronbach's alpha for the SF-36V physical functioning subscale was 0.90 for this sample.

#### Self-Rated Health (SRH)

The SF-36V includes six items relating to SRH—overall health now (“In general, would you say your health is excellent, very good, good, fair or poor?”), overall health compared to a year ago (much better, somewhat better, about the same, somewhat better, much worse), and four items that yield a general health index (e.g., “I am as healthy as anyone I know.”) Because two different response formats were used, raw scores were converted to z-scores. The internal consistency of the six items, estimated using Cronbach's alpha, was found to be 0.64, which was judged to be too low. When one question—“Compared to 1 year ago, how you would rate your health in general now?”—was dropped, alpha rose to 0.77. Therefore, the mean of the five z-scored items was used to operationally define SRH.

#### Competency in Everyday Functioning

The Patient Competency Rating Scale (PCRS) (91) elicits patients' self-rated competency in 30 specific activities that TBI commonly impairs (91). Thus, the PCRS was developed specifically for TBI and has been used with military TBI populations (92). The 30 items encompass four domains: cognitive, physical, emotional, and interpersonal, although in some study samples, a single factor loads both emotional and interpersonal items, creating a 3-factor structure (76, 85). The stem question is worded, “How much of a problem have you had (in the past month) in…”? Participants respond using a 5-point Likert Scale (1 = cannot do; 2 = very difficult to do; 3 = can do with some difficulty; 4 = fairly easy to do; 5 = can do with ease). The PCRS has demonstrated good internal consistency and predictive validity for return to work, community integration, and global functioning 1-year post-injury. Cronbach's alpha for the present sample was 0.92 for the overall scale, 0.81 for physical functioning, 0.84 for cognitive functioning, and 0.87 for emotional/interpersonal functioning. In the present analysis, the emotional functioning items were not used as they would be confounded with depressive symptoms.

Physical functioning was thus operationalized in two ways, using the Medical Outcomes Short Form Health Survey for veterans (SF-36V) and the Patient Competency Rating Scale (PCRS). Both are well-established measures of self-rated everyday functioning with different foci: the PCRS was designed specifically for TBI deficits, whereas the SF-36V was designed to capture everyday functioning in the general population. Importantly, both have a physical functional limitations component. Table 1 presents the physical functioning items in the two scales. The overlap between these subscales dictated an analytical strategy using two regression models, described below.

**Table 1:** Two physical functioning measures: the Medical Outcomes Survey Short Form 36 vs. the Patient Competency Rating Scale (PCRS).

	**Medical Outcomes Survey—Short Form—Veterans** **(SF-36) physical functioning scales**	**Patient Competency Rating Scale (PCRS)**
Stem question	How limited are you in…?	How much of a problem have you had (in the past month) in… ?
Response format	A lot, a little, not at all	Cannot do, Very difficult, Somewhat difficult, Fairly easy, Can do with ease
**ITEMS**		
1.	Vigorous activities, such as running, lifting heavy objects, participating in strenuous sports	Preparing your own meals
2.	Moderate activities, such as moving a table, pushing a vacuum cleaner, bowling, or playing golf	Dressing yourself
3.	Lifting or carrying groceries	Taking care of your personal hygiene
4.	Climbing several flights of stairs	Washing the dishes
5.	Climbing one flight of stairs	Doing the laundry
6.	Bending, kneeling, or stooping	
7.	Walking more than a mile	
8.	Walking several hundred yards	
9.	Walking one hundred yards	
10.	Bathing or dressing yourself	

#### Depressive Symptomatology

Depressive symptomatology was assessed with the 10-item Center for Epidemiologic Studies Depression Scale—(CES-D) short form (93). This screening instrument assessed the frequency of each symptom in the past week on a 0 (never or rarely) to 3 (every day) Likert scale, producing a possible range of 0–30, with higher scores reflecting higher depressive symptomatology. A cut-off score of 10 or higher indicates the presence of clinically significant depressive symptoms. The CES-D short form has well-established psychometric properties. In a large national sample of Operation Enduring Freedom (OEF, i.e., Afghanistan war) and Operation Iraqi Freedom (OIF, i.e., Iraq war) veterans (93), internal consistency of the CES-short from was reported as 0.91. Cronbach's alpha for the present sample was 0.85.

### Procedure

The Institutional Review Board of the Corporal Michael J. Crescenz Veterans Affairs Medical Center VA Medical Center approved the study. Participants were interviewed in their homes by a trained interviewer. This interview provided information about participants' sociodemographic characteristics (including employment status), depression, and everyday functioning, as well as other background measures germane to the study (e.g., community reintegration). Comorbidities, TBI severity, and time post injury were obtained through a review of CPRS.

### Data Analyses

Bivariate relationships between each predictor and employment status were tested using independent measures *t*-tests or Chi-square tests, as appropriate. The potential predictors were years since most recent TBI, severity of most recent TBI, comorbidities (PTSD and pain), the SF-36V physical functioning subscale, and three PCRS domains (physical, cognitive, and interpersonal functioning), and depressive symptoms. The variables that revealed significant bivariate associations with employment status (*p* < 0.05) were selected as predictors in the subsequent analyses.

Hierarchical binary logistic multiple regression was used to test mediation. Although newer and more robust approaches such as structural equation modeling (SEM) to test mediation exist, (39, 94) hierarchical multiple regression was appropriate given the present study's smaller sample size (94–96). The data satisfied Baron and Kenny's (39) criteria to establish mediation (i.e., significant bivariate intercorrelations among independent, dependent, and mediating variables) (see below). Because employment status was a two-level dependent variable, binary logistic regression was used. The general analytic plan was to enter predictors in order of their modifiability from least to most, with background injury-related characteristics (e.g., time post injury) first, comorbidities next (PTSD, pain), and finally TBI sequelae, with physical health, functioning, and SRH in one block, and emotional sequelae (depressive symptoms) in the final block. Depressive symptoms were entered separately and after physical health/functioning because past research has cited depression as a mediator of other health conditions.

It is important to note that, for some study participants, higher education, rather than employment, may have been more important than employment. The GI Bill of Rights, a U.S. military benefit since 1944, provides financial support for college, graduate school, and training programs for veterans (97), and many veterans take advantage of this education benefit. In our study, 17 participants identified themselves as full-time students. To allow for the possibility that education may have been these veterans' primary goals (rather than employment), we conducted the regression analyses both with and without their data. SPSS version 20 was used for all analyses.

## Results

### Description of Sample

Only about one-third of the veterans were employed, as shown in Table 1. None were voluntarily retired. Their mean age was 42 years, ranging from 23 to 67 years. Most (92%) were male. About 58% were white, 35% were Black, and 14% reported themselves as Hispanic or Latino. More than two-thirds were married, and 76% had children. Table 2 also presents data on veterans' war cohort, source of TBI, number of TBI-incidents, TBI severity, comorbidities (e.g., PTSD), time since most recent TBI, and prevalence of major TBI-related sequelae. Most participants were veterans from Operation Iraqi Freedom (OIF), followed by those from Operation Enduring Freedom (OEF, i.e., the Afghan war). Time since the most recent TBI (time post injury) ranged from 1 to 45 years (for a Vietnam War veteran), with a mean of nearly 10 years. Almost one-third had experienced both blast and mechanical injuries. Thirty-six percent had experienced more than four TBIs, whereas only one-third reported a single TBI incident. Approximately 65% had a PTSD diagnosis, and 60% had a depression diagnosis documented in CPRS. Table 2 presents these sample characteristics.

**Table 2:** Sociodemographic, medical, and military characteristics of the sample (*n* = 83).

	**Percent (*n*)**	**Mean (*SD*)/range**
Age		40.13 (13.20)/23–67 years
Gender (% male)	91.9 (76)	
**EDUCATION**		
Less than high school degree	6.0 (5)	
High school degree or GED	24.1(20)	
Some college	45.8 (38)	
College degree	16.9 (14)	
Postdoctoral degree	7.2 (6)	
Marital status (% married)	69.9 (58)	
Financial difficulty^*^		1.62 (1.09)/0–3
Employed	34.9 (29)	
**RACE**		
White	57.8 (48)	
Black	34.9 (29)	
Native Amer.	2.4 (2)	
Asian	1.2 (1)	
No primary/other	3.6 (3)	
Hispanic/Latino	14.0 (12)	
**SEVERITY**		
Mild	68.7 (57)	
Moderate-severe	31.1(26)	
**WAR COHORT^3^**		
OIF (Iraq)	61.4 (51)	
OEF (Afghanistan)	22.9 (19)	
Both OIF and OEF	10.5 (9)	
Prior to OEF/OIF	28.8 (24)	
Years since most recent TBI		9.99(11.09)/1.0–45.4 years
PTSD diagnosis	65.1 (54)	
Depression diagnosis	50.6 (42)	

* Difficulty paying for the basics such as housing, rated on a scale from 0 (not at all difficult) to 3 (extremely difficult).

3 *Does not sum to 100% because some veterans served in multiple war cohorts*.

### Bivariate Associations With Employment Status

Tests of zero-order associations of sociodemographic characteristics and comorbidities with employment status revealed no associations. Employment status was found to be significantly associated with the pain index of the SF-36V; physical functioning as measured by both the PCRS and the SF-36V; the SRH index; the cognitive functioning factor of the PCRS; and depressive symptoms. Therefore, only these variables were used in the logistic regression analyses. Table 3 presents these bivariate findings.

**Table 3:** Bivariate associations between employment status and predictor variables: results of *t*-tests or Chi-square tests.

**Predictor**	**Employed** **mean (SD) or % (n)**	**Unemployed** **mean (SD) or % (n)**	**t (df)**	**Chi^**2**^ (df)/** **Fisher's exact**	***p***
Age	37.03 (12.67)	41.72 (13.03)	1.578 (81)		0.118
Sex [male (72)]	34.2 (26)	65.8 (50)		.	0.691
Race [White (46)]	33.3 (16)	66.7 (32)		0.129 (1)	0.719
Hispanic ethnicity (11)	36.4 (4)	63.6 (7)		0.011 (1)	0.915
Financial difficulty	1.45 (0.10)	1.69 (1.11)	0.961 (81)		0.339
Marital status (married(56)]	36.2 (21)	63.8 (37)		0.061 (1)	0.804
Education [> high school (56)]	39.7 (23)	60.3 (35)		1.917 (1)	0.166
PTSD [diagnosis present (51)]	29.6 (16)	70.4 (38)		1.884 (1)	0.170
Pain [Diagnosis present (53)]	32.1 (18)	67.9 (38)		0.916 (1)	0.339
Tinnitus [Diagnosis present (21)]	47.6 (10)	52.4 (11)		1.853(1)	0.173
Photosensitivity [Diagnosis present (17)]	23.5 (4)	76.5 (13)		1.344 (1)	0.246
Years since TBI	7.43 (9.22)	11.36 (11.82)	1.56 (81)		0.124
TBI severity [mTBI (56)]	40.4 (23)	59.6 (20)		2.344 (1)	0.126
Depressive Symptoms (CES-D)	15.00 (6.30)	20.17 (5.50)	3.88 (81)		< 0.001
**SF-36V (PHYSICAL HEALTH/FUNCTIONING COMPONENTS)**					
Physical functioning	5.28 (4.61)	9.65 (4.72)	4.06 (81)		0001
Pain severity	2.79 (1.05)	3.43 (1.25)	2.32 (81)		0.023
Extent pain interferes with work^*^	1.93 (1.36)	2.65 (1.20)	2.48 (81)		0.015
Pain Composite	2.36 (1.13)	3.04 (1.14)	2.57 (81)		0.012
SELF-RATED FUNCTIONING (PCRS)^**^					
Cognitive	3.00 (0.76)	2.71 (0.54)	2.00 (81)		0.049
Interpersonal	3.32 (0.82)	3.09 (0.70)	1.37 (81)		0.174
Physical	4.24 (0.45)	3.12 (81)	3.12 (81)		0.002

* Mean of pain severity and pain interference scores.

** *Patient Competency Rating Scale, omitting emotion items to avoid confounding with depressive symptoms*.

### Bivariate Associations Among the Predictors

An assumption of multiple regression mediation analyses is that independent variables and mediators must be correlated with the dependent variable and with each other. In these data, the requirement was met. CES-D scores were found to be correlated with both SF-36V physical functioning (*r* = 0.42, *p* < 0.0001) and the PCRS physical functioning measure (mean = −0.56, *p* < 0.0001). The pain index was also strongly associated with both SF-36V (*r* = 0.61, *p* < 0.0001) and the PCRS measures of physical functioning (*r* = −0.49, *p* < 0.0001). All had bivariate associations with employment status (Table 2).

### Logistic Regressions

The pain index (a comorbidity) was entered on block 1; physical functioning, cognitive functioning, and SRH on block 2; and depressive symptoms on block 3. Because physical functioning was operationally defined in two ways, using the SF-36V and the PCRS physical functioning measures, a separate regression was conducted for each physical functioning measure.

### Regression Using SF-36V Measure of Physical Functioning

**Figure 1:**
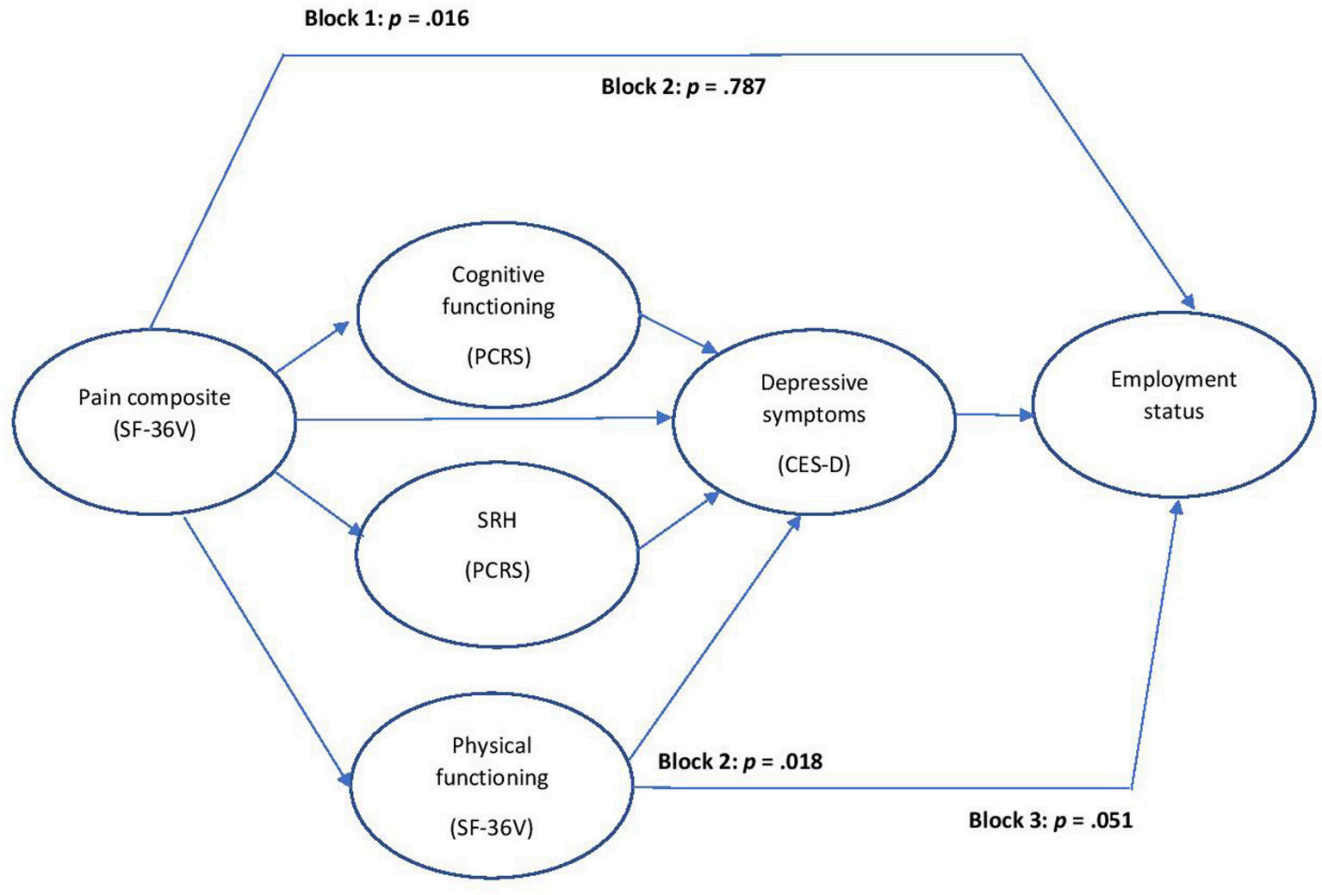
Mediation effects: pain effect mediated by physical/health functioning (at Block 2); physical functioning effect mediated by depressed symptoms (at Block 3).

On block 1, the pain index revealed an association with employment status, but this association became nonsignificant when the physical health/functioning variables were entered on block 2. Among those block 2 predictors, only physical functioning demonstrated an independent association with employment status. On block 3, the entry of depressive symptoms significantly attenuated the physical functioning effect, leaving depressive symptoms as the sole significant predictor. Table 4 presents these findings. Figure 1 displays the mediation effects.

**Table 4:** Binary logistic regression results: employment status' association with predictors (using SF-36V definition of physical functioning), demonstrating mediation of pain effects by physical health/functioning and mediation of physical functioning by depressive symptoms (Nagelkerke *R*^2^ = 0.313, *p* = 0.001).

					**95% CI**	
	**B (Std error)**	**Wald (df)**	***p***	**Exp(B)**	**Lower**	**Upper**
**BLOCK 1**						
Pain (severity and interference with normal work)	−0.508 (0.211)	5.820 (1)	0.016	0.602	0.399	0.903
**BLOCK 2**						
Pain (severity and interference with normal work)	−0.078 (0.287)	0.073 (1)	0.787	0.897	0.517	1.556
Self-rated health	−0.071 (0.489)	0.021 (1)	0.885	0.752	0.248	2.278
Cognitive functioning (PCRS)	0.261 (0.431)	0.368 (1)	0.544	1.322	0.569	3.073
Physical functioning (SF-36V)	−0.176 (0.075)	5.578 (1)	0.018	0.833	0.721	0.962
**BLOCK 3**						
Pain (severity and interference with normal work)	−0.038 (0.291)	0.017 (1)	0.897	0.923	0.526	1.619
Self-rated health	0.097 (0.510)	0.036 (1)	0.850	0.879	0.282	2.743
Cognitive functioning (PCRS)	−0.451 (0.548)	0.679(1)	0.410	0.669	0.230	1.740
Physical functioning (SF-36V)	−0.148 (0.076)	3.821 (1)	0.051	0.854	0.738	0.989
Depressive symptoms	−0.133 (0.057)	5.445 (1)	0.020	0.878	0.786	0.981

### Regression Using the PCRS Measure of Physical Functioning

As with the previous regression model, pain (block 1) lost its predictive ability when physical health/functioning variables were entered on block 2, but none of these individual predictors independently predicted employment. Thus, the PCRS measure of physical functioning did not predict employment status, as the SF-36V definition had. Only depressive symptoms, entered on block 3, demonstrated a significant independent contribution to employment status. Table 5 presents these regression findings.

**Table 5:** Binary logistic regression results: employment status association with predictors (using PCRS measure of physical functioning), demonstrating mediation of pain effect by physical health/functioning (Nagelkerke *R*^2^ = 0.259, *p* = 0.001).

					**95% CI**	
	**B (Std error)**	**Wald (df)**	***p***	**Exp(B)**	**Lower**	**Upper**
**BLOCK 1**						
Pain (severity and interference with normal work)	−0.508 (0.211)	5.820 (1)	0.016	0.602	0.398	0.905
**BLOCK 2**						
Pain (severity and interference with normal work)	0.178 (0.265)	0.450 (1)	0.503	0.779	0.471	1.287
Self-rated health	0.326 (0.437)	0.557 (1)	0.455	1.126	0.412	3.076
Cognitive functioning (PCRS)	0.199 (0.460)	0.187(1)	0.665	1.265	0.517	3.095
Physical functioning (PCRS)	0.822 (0.513)	2.569 (1)	0.109	2.263	0.838	6.113
**BLOCK 3**						
Pain (severity and interference with normal work)	−0.176 (0.283)	0.389 (1)	0.533	0.767	0.446	1.321
Self-rated health	0.411 (0.465)	0.780 (1)	0.377	1.184	0.409	3.426
Cognitive functioning (PCRS)	−0.438 (0.554)	0.626 (1)	0.429	0.693	0.239	2.010
Physical functioning (PCRS)	0.475 (0.569)	0.695 (1)	0.405	1.598	0.531	4.806
Depressive symptoms	−0.137 (0.058)	5.497 (1)	0.019	0.875	0.781	0.980

Regression analyses, performed without the 17 cases of full-time students, produced results not substantially different from the analyses using the full sample.

## Discussion

Pain was a significant predictor of employment status, but its effect was attenuated by the physical health and functioning variables—among which physical functioning, as measured by the SF-36V, was the sole significant predictor in block 2. This physical functioning effect in turn was attenuated when depressive symptom scores were entered into the regression model. Mediation was total. These findings illustrate the value of mediation analyses in yielding insights into the contributions of predictors of TBI outcomes, in this case employment status. Mediation analyses have particular utility for tertiary prevention of poor TBI outcomes, an important aim in light of the many TBI patients left with chronic TBI symptoms after post-acute rehabilitation ends.

For the PCRS measure of physical functioning, this effect was not demonstrated. Neither the PCRS measures of cognitive or physical functioning, although both had bivariate association, made independent contributions to employment status. By contrast, it is interesting to note the utility of the SF-36V measures of health and functioning, especially bearing in mind that this tool was not originally intended for a TBI population. Inspection of the two measures (Table 1) suggests that the items in the PCRS measure closely correspond to instrumental activities of daily living (i.e., self-care activities), whereas the SF-36V items address a broader range of basic physical activities including walking, bending, lifting, and climbing stairs. The SF-36V measure also included more items overall. In addition to its measure of physical functioning, SF-36V measures of pain and SRH were associated with employment status. These results highlight the value of the SF-36V for TBI research.

Considering that most of the sample had an mTBI, the finding that limitations in physical functioning predicted employment status was somewhat surprising. Yet, many previous TBI studies have identified physical functioning limitations as a predictor of employment status. The fact that this variable as measured by the SF-36V and the CPRS showed significant bivariate associations with employment argues against measurement error as an explanation. Nevertheless, the possibility exists that non-TBI injuries in this population, drawn from a polytrauma population, may have accounted for the physical functioning findings. A replication of this effect using a civilian sample with less polytrauma should address this interesting question.

Although employment may be thought of as a component of CR and even as a possible proxy for it, it should be noted that in the present data set, CR and employment status were not strongly associated (*r* = 0.21, *p* = 0.054). Therefore, the findings of mediation of physical functioning effects on employment outcomes by depressive symptoms contributes evidence for the robustness of depression's effect.

In the present sample of military veterans with TBI, no sociodemographic characteristics were associated with employment status. This differs from findings of many studies of civilian TBI. It is possible that, because of participants' shared backgrounds in the military, some sociodemographic characteristics (i.e., previous employment, sex, number of years of education) varied less than would be the case in civilian TBI samples. Similarly, TBI severity was not associated with employment status, possibly reflecting the predominantly mild or moderate severity status of this sample.

Cognitive functioning, defined using the PCRS cognitive domain, was also not found to be a predictor of employment status. This may reflect the fact that this variable was a self-rated measure, rather than based on neuropsychological test scores, which some studies have found to be predictive of employment outcomes (12, 24). The fact that the present sample included few patients with severe TBI could also help account for the absence of effects for cognitive functioning.

Findings underscore the central role of depression in the employment status of veterans with TBI. They echo earlier research (58) that showed depressive symptoms mediated effects of both physical functioning and PTSD diagnosis on community reintegration in veterans with TBI.

### Study Limitations

Although hierarchical multiple regression was appropriate given the study's relatively small sample size, SEM or other methods might be more powerful for testing mediation in larger samples (98). The mediating role of depression in employment status has not been identified in previous TBI research, and thus it warrants further examination in future studies having larger and more diverse samples. A larger sample might demonstrate statistically significant effects for the mediated predictors, revealing partial mediation for some of the predictors. Our findings should be considered preliminary until they can be replicated in such samples using different mediation methods.

Because multiple regression analyses are correlational, inferences about the direction of causality should not be drawn. For example, unemployment may itself be a cause of depression, rather than an effect of it. Furthermore, depression may have existed prior to the TBI, rather than being an effect, and may have contributed to functional limitations. The present investigation did not have access to information about time of onset for depression. These possibilities complicate interpretation of the findings. Thus, an interesting issue for future research might be whether time of onset of depression makes a difference in findings. In addition, the study's cross-sectional design precludes interpretation of temporal relationships, which might be addressed with longitudinal data. Most of our sample had experienced mild TBI with persistent post-concussive symptoms, and 12% had mild to moderate TBI. This preponderance of milder TBI cases may help account for differences from earlier studies with patients with primarily moderate to severe TBI reporting severity as a predictor of work outcomes (22, 23).

The generalizability of study findings may be limited because this sample consisted of veterans enrolled in outpatient services at a VA rehabilitation service. Future research should include samples of civilians with TBI, as well as veterans with TBI who are *not* using VA services. In our sample, 89% had clinically significant levels of depressive symptoms, which is likely to be higher than in civilian TBI populations. Nevertheless, TBI populations do have a high prevalence of depressive symptoms and depression diagnoses (99).

Questions remains about other mediators and specifically whether variables entered earlier in the model (e.g., pain, here treated as a comorbidity and therefore entered on block 1) may themselves mediate effects of variables entered later. Such questions would rely on theoretical rationales regarding causality and may be interesting directions for further research.

### Other Directions for Future Research

Moderation analyses should also be more routinely employed in research on employment and TBI. Whereas mediation speaks to how or why relationships occur, moderation reflects the direction and/or strength of the relation between an independent and a dependent variable (39). Usually represented as interactions, moderators may shed light on ways in which predictors interact to produce effects. Given the size and richness of research on employment status and TBI, researchers should employ more moderation approaches.

Some participants were in school, utilizing their GI Bill education benefits, rather than employed. This was addressed by conducting the analyses without data from those in school fulltime and not working. The fact that education, rather than employment, may be a primary rehabilitation goal may be an issue for civilian samples as well, given that many young school-age adults sustain TBI. Research with large civilian TBI samples could examine whether college/training attendance have similar predictors as employment status.

### Clinical and Research Implications

TBI rehabilitation often aims to return persons with TBI to meaningful employment, which helps them restore previous social roles, promotes socialization, improves psychological well-being, and increases opportunities for building relationships. All of these are part of reintegrating into the community, the premier goal of rehabilitation (100). The ability to work also has implications for the family and its financial status. Financial difficulty has been shown to predict depressive symptoms in family caregivers of veterans with TBI (101). The patient's inability to work thus may also have effects on caregiver well-being.

Study findings underscore the value of mediational analyses in shedding light on how risk factors “work together” to affect outcomes of interest (102). They argue that mediational analyses should be used more routinely in research on important TBI outcomes such as employment. Because depression is amenable to treatment, its recognition as a mediator provides opportunities to influence important health outcomes, particularly as a means of tertiary prevention. The present findings suggest that the negative effects of pain and/or physical functioning limitations on employment could be mitigated by more effective treatment of depression. Therefore, routine assessment of depressive symptoms and aggressive treatment of depression should promote the success of rehabilitation in improving employment outcomes for individuals with TBI.

## Ethics Statement

The Institutional Review Board of the Corporal Michael J. Crescenz Veterans Affairs Medical Center in Philadelphia, PA approved the study and the study protocol. Informed consent was obtained using a Veterans Affairs written consent form for human participants. This study was carried out in accordance with the requirements of the Institutional Review Board of the Corporal Michael J. Crescenz Veterans Affairs Medical Center with written informed consent from all subjects. All subjects gave written informed consent in accordance with the Declaration of Helsinki.

## Author Contributions

All authors listed have made a substantial, direct and intellectual contribution to the work, and approved it for publication.

### Conflict of Interest Statement

The authors declare that the research was conducted in the absence of any commercial or financial relationships that could be construed as a potential conflict of interest.
